# Feeding children with autism in South Africa: The teachers’ perspectives

**DOI:** 10.4102/ajod.v12i0.1252

**Published:** 2023-11-02

**Authors:** Skye N. Adams, Nthabiseng Matsimela

**Affiliations:** 1 Department of Speech Pathology and Audiology, Faculty of Humanities, University of the Witwatersrand, Johannesburg, South Africa

**Keywords:** autism, classroom, feeding support, teachers, South Africa

## Abstract

**Background:**

Over 80% of children diagnosed with autism spectrum disorders (autism) exhibit disruptive behaviours during mealtimes, highlighting the need for personalised care. In South Africa, teachers often take on the responsibility of feeding due to resource constraints and the time children spend at school. Moreover, children with autism have unique and individualised feeding requirements, which many teachers may not have the necessary training or skills to address adequately.

**Objectives:**

To explore the ways in which teachers of autistic children manage feeding difficulties in the classroom.

**Method:**

A qualitative research design was employed using semi-structured interviews. Eight teachers were interviewed on feeding autistic children between the ages of 3 years - 9 years in Johannesburg, South Africa. Data were transcribed and analysed using thematic analysis.

**Results:**

The findings revealed that teachers encountered distinct challenges when it came to feeding autistic children in the classroom, particularly concerning the management of associated feeding difficulties. Teachers employed several strategies to encourage eating in the classroom setting including: (1) bolus modification, (2) behaviour modelling, (3) positive reinforcement and (4) offering choices and alternatives.

**Conclusion:**

The study concludes the need for specialised support and training for teachers to address the individualised feeding needs of children with autism. Implementing targeted interventions and providing resources for teachers could enhance their abilities to effectively support children with autism during mealtimes and promote a more inclusive classroom environment.

**Contribution:**

This study highlighted the importance of including the teacher in the multidisciplinary team when managing the feeding challenges in children with autism.

## Introduction

Autism spectrum disorder (autism) is a complex neurodevelopmental disability characterised by deficits in social communication and interaction, alongside restricted and repetitive patterns of behaviour, interests or activities (Rapin & Tuchman 2008). Approximately 89% of the autism population may present with food selectivity, sensory processing difficulties and tactile defensive behaviours that can negatively impact growth, development and nutritional intake and if left untreated may become chronic (Nadon et al. 2011; Seiverling et al. 2018; Zhu & Dalby-Payne 2019). Children with autism often experience feeding difficulties, such as food sensitivities and aversions to certain textures, temperatures or smells, which are usually evident in early childhood (Ausderau & Juarez 2013; Viviers et al. 2020). Consequently, caregivers of young children with autism and associated feeding difficulties often resort to employing targeted interventions and strategies to address and improve these challenges (Ausderau et al. 2019; Ledford, Whiteside & Severini 2018; Schaaf et al. 2014). However, as children begin school, the responsibility of feeding is no longer primarily the caregiver’s concern but the teachers’. Currently, there is a paucity of research on teacher’s utilisation of feeding strategies for children with autism (Knox et al. 2012).

In this article, the authors shed light on the complexities surrounding feeding children with autism and associated feeding difficulties, specifically from the viewpoint of their teachers. While the experiences of these teachers are similar to those of teachers globally, the authors aim to gain insights from an Afrocentric perspective by incorporating culturally and contextually relevant feeding strategies. Upon starting school, teachers assume the responsibility of providing food and managing mealtimes for the child, often spending more time with them than their caregivers during the week, approximately five out of seven days, between six and eight hours each day (Knox et al. 2012; Marsh et al. 2017). A typical South African classroom of children with autism usually involves a teacher and a teaching assistant (Donohue & Bornman 2015). The role of the teacher is to facilitate all teaching and assistant to support the teacher and specific learners. In South Africa, inclusive education has faced challenges, leading many children with autism to attend specialised schools (Nthibeli, Griffiths & Bekker 2022; Sumbane et al. 2023). Children in these classes present with a range of needs, attributes, experiences and abilities, with children and teachers coming from a variety of cultural, religious and linguistic backgrounds (Forghani-Arani, Cerna & Bannon 2019). Despite this diversity in the classroom, very little is known about the ways in which teachers navigate this space when dealing with feeding difficulties in this environment.

Many children with autism will require specialised care to manage their feeding challenges and may require consultation with healthcare professionals such as speech-language therapists (SLTs). However, there is a shortage of SLTs in South Africa, and with the high prevalence of feeding difficulties in children with autism, teachers are often required to provide supportive feeding strategies without the assistance of the SLTs or availability of resources (Abubakar, Ssewanyana & Newton 2016; De Vries 2016; Sumbane et al. 2023). This is in contrast to other countries such as Australia or France, where teacher support is available through government policies and guidelines that call for further teacher education and allocation of resources to better support children with autism (Boujut et al. 2016; Clark et al. 2020). Research where teachers have high levels of support and training with autism has indicated that they are less burdened and experience less negative emotional responses (Boujut et al. 2016; Clark et al. 2020). Thus, there is a need to understand the ways in which teachers manage the feeding difficulties in children with autism, the supports and resources available to them, and the ways in which the strategies are employed within a school setting.

Effectively managing feeding difficulties in children with autism demands consistency, time and effort and cannot be achieved in isolation or confined to one context only (e.g. school, therapy or home) (Kerzner et al. 2015; Van Den Hoek-Engel et al. 2017). Seiverling et al. (2012) investigated the impact of a home-based parent behavioural skills training programme aimed at enhancing the treatment of food selectivity in children with autism. The behavioural skills taught to parents encompassed taste exposure, escape extinction (which entails not allowing the child to avoid disliked foods) and fading (gradually reducing the level of assistance required for a specific task). The findings demonstrated a positive outcome in terms of increased food consumption among all children in the study after undergoing the treatment. However, it was evident that these strategies were not already a natural part of their behaviour, and there were limitations in integrating these behaviour techniques into family mealtimes. The study emphasised the necessity for parents to receive targeted training to effectively employ these techniques and seamlessly integrate them into their mealtime routines and rituals. Furthermore, the study emphasised the need to tailor interventions to the family’s specific routine, beliefs and rituals (Bornstein 2013). Studies have shown that feeding management may prove ineffective if it fails to consider and respect the specific needs, cultures, beliefs and values of the families involved (Adams, Dadabhay & Neille 2021; Ausderau et al. 2019; Ochs & Shohet 2006; Suarez, Atchison & Lagerwey 2014). Although this is true for families, teachers may hold their own beliefs and values that they bring to the classroom, and the ways in which these are incorporated require interrogation. Currently there is limited research around the specific interventions that teachers working with children with autism can implement, specifically around managing feeding and nutritional challenges in the classroom. The results of this study are expected to make a significant and pertinent contribution to the understanding of autism in both the South African context and on a global scale. The study will provide insight into the strategies used by teachers to manage feeding difficulties in children with autism in South African classrooms, considering the lack of SLTs and available resources for support.

## Research methods and design

A qualitative methodology using semi-structured interviews was employed to gain a better understanding of the ways in which teachers manage feeding difficulties of children with autism in the classroom. Teachers were purposively sampled according to the following criteria: (1) previous or current experience feeding children with autism, (2) currently working at an autism school in Johannesburg. Teachers had to be working with children: (1) who had primary diagnosis of autism and (2) who were between the ages of 3 years and 9 years. Teachers were not required to have any specific length of experience but had to be working at the school for a minimum of six months. The age range of the child was chosen to approximate children who may experience feeding challenges and those of school age for homogeneity of the sample.

### Ethical considerations

Prior to conducting the interviews and after obtaining clearance from the University of the Witwatersrand Health Research Ethics Committee – Non-Medical (HREC Non-Medical) (STA_2020_07), a pilot study was conducted. Thereafter, letters of permission were sent to the school principals of four government autism schools in Johannesburg and received responses from two schools which had granted permission for participation. Permission from the site was received from them prior to commencement of the interviews. The principal sent a message to all teachers at their schools and asked them to identify teachers who met the inclusion criteria. Details of the study and informed consent letters were circulated and teachers were asked to contact the researcher if interested. Following the recruitment of participants and the coronavirus disease 2019 (COVID-19) lockdown that was implemented in March 2020, all interviews were conducted telephonically after verbal consent was obtained. All study participants were informed of their right to refuse participation and to stop the interview at any time. All personal information such as names and specific locations were left out of the transcription to ensure anonymity and confidentiality.

### Participants

Eight teachers participated in the research with previous and current experience ranging from 8 months to 8 years. Participants had different home languages, although all were fluent in English and the medium of instruction at both schools is English. The age of participants ranged from 25 years to 45 years, and all participants were female. All children in the classes had a formal diagnosis of autism and their ages ranged from 3 years to 9 years. Participants’ descriptions are provided in Table 1.

**TABLE 1:** Participants’ demographics.

Teacher	Age (years)	Home language	Sex	*N* = years or months experience
1	31	isiZulu	F	8 months
2	27	Sotho	F	10 months
3	45	Sotho	F	8 years
4	31	English	F	7 years
5	25	English	F	3 years
6	45	Afrikaans	F	6 years
7	28	Tswana	F	4 years
8	34	isiZulu	F	3 years

### Data collection

Data were collected during April–June 2020. A semi-structured interview questionnaire (Appendix 1) was used to guide the interviews. Online semi-structured interviews were conducted with the participants at times convenient for them in their preferred language, either English or isiZulu, through telephonic interviews. Participants did make use of code-switching between English and isiZulu, which the researcher was able to understand, translate and transcribe into English. The semi-structured interviews provided an opportunity for open and in-depth discussions. Each interview, lasting approximately 1 h, was audio-recorded and transcribed verbatim. As a result of COVID-19 restrictions at the time, all interviews were conducted over the phone following the initial contact.

### Data analysis

The collected data underwent inductive thematic analysis. All transcripts were imported into the NVivo computer programme for coding and text content analysis. The framework method was employed to systematically categorise and code the interview transcripts, ensuring a rigorous and high-quality analytical process (Ritchie, Spencer & O’Connor 2003; Saldaña 2013). At the outset, codes were formulated based on existing literature regarding teachers’ experiences of feeding children with autism, employing a deductive approach. The framework was then piloted on two interviews separately by the two researchers, and any discrepancies or differences were resolved before applying the framework to all transcripts. In addition, during coding, the framework was supplemented with codes generated from inductive analysis. This comprehensive framework was then used for all interviews. Throughout the research process, the researchers engaged in reflexive note-taking to acknowledge and record researcher bias. The second author independently validated the themes and analysed the transcripts, leading to a preliminary intercoder agreement. Following this agreement, all themes and labels were thoroughly discussed and mutually agreed upon by both authors. The process of inductive thematic saturation was achieved after analysing all the interviews.

To ensure data trustworthiness, the researcher explored five critical components of credibility, transferability, dependability, confirmability and reflexivity (Anney 2014). Several measures were put in place to ensure rigour and trustworthiness of findings. Credibility was determined through member checking. To maximise specific data relative to the context in which it was collected and to establish the likeliness of transferability, the researcher used a purposeful sampling strategy. Dependability was achieved by providing detailed descriptions of the data collection and data analysis processes to replicate the study. To ensure confirmability, the researcher diligently documented procedures for cross-verifying and validating data throughout the study. Additionally, to maintain reflexivity and avoid any potential bias, the researcher consistently and manually took notes using a research journal during the research process.

## Results

Teachers in this study utilised a variety of strategies to manage feeding and related difficulties although they were implemented flexibly and when necessary by teachers. All teachers explained that a typical feeding time in the classroom was approximately 20 min – 30 min. In addition, majority of children had breakfast, morning snacks, lunch (before going out to play) and afternoon snacks in the classroom. Teachers reported classroom sizes of between 6 and 12 children. Parents provided the food for their child in a lunchbox, and the teachers would then give it to them throughout the day, depending on what meal it was. All strategies targeted increased food acceptance and food intake of both familiar and unfamiliar food items (unfamiliar foods were often given to children from their peers’ lunchbox if they showed interest). Four strategies emerged as reported by the teachers, including (most to least prevalent): (1) bolus modification, (2) behaviour modelling, (3) positive reinforcement and (4) offering choices and alternatives. It is important to note that teachers implemented these strategies to all children, regardless of their age. All of these are discussed in greater detail in the following sections.

### Bolus modification

Bolus modification was the predominant strategy used by teachers for children of all ages in this study. All teachers spoke of having to cut food into smaller bite-size pieces, mashing food and feeding the learner smaller bits at a time as reported by Teacher 3, ‘I’d cut down the hotdog into smaller pieces and then give him piece by piece and then eventually I’ll … like start giving him bigger pieces’ and Teacher 8, ‘Remember when I said I had to cut the food small? Having to cut the food small or sometimes I need to mash the food’. Another teacher spoke about having to modify the food by removing all the beans in the dish so the child would eat their food:

‘There is a child who can eat samp^[1]^ but only prefers white samp without sugar beans, so you have to remove all beans and if you leave even one in the plate or try to hide it and they don’t see it. When you feed him, he will still feel it in his mouth and you will just see him running to the bin to spit out the food.’ (Teacher 1, age 31, isiZulu)

Teacher 1 went on to explain that often it was the teacher’s responsibility to make these changes and often had to rely on information from the child’s parents (if available), their teaching assistance and their own knowledge and experience of working with children with autism:

‘Some parents would communicate and some will not. And uh … assistants … in the class would say the child would eat 123 and comfortable with certain foods. And uhm … I also knew how autism kids behave from theory because I did a course in autism so I had background information.’ (Teacher 1, age 31, isiZulu)

Teacher 3 stated the importance of knowing the children in your class and their feeding preferences, ‘So it is really just a matter of knowing the child and preferences in the class’. However, teachers still found themselves having to improvise and rely on their own understanding of autism, as well as previous experience. Although some teachers reported on additional training for autism (as reported by Teacher 1 earlier), many teachers did not receive any additional training and were required to learn while in the class, as Teacher 4 explained:

‘I just try to improvise where I can to assist them so basically; I didn’t get so much training or input from outside but basically everything happens in the class. Most of the teachers, are the learner themselves.’ (Teacher 4, age 31, English)

After modifying the bolus either by changing the texture, size or even type of food provided, all teachers reported that there was increased food acceptance. Teachers also emphasised the need to never force-feed a child as that did not result in any positive feeding outcomes, but to instead try and provide different modifications to the bolus and work on a more graded approach as expressed by Teacher 3:

‘So we will slowly present different types of meals but we don’t force them … So we just slowly introduce different types of textures and colors. So they will start by feeling and familiarise themselves with the type and smell before they start eating and then they are willing to try.’ (Teacher 3, age 45, Sotho)

Thus, the process of working on bolus modifications proved successful in the classroom, as reported by teachers. They highlighted factors that facilitated increased food acceptance, including different ways in which to modify the food and refraining from force-feeding the children.

### Behaviour modelling

Six teachers reported using behaviour modelling to address both food-related and behavioural problems. Behavioural modelling was performed by having the teachers or teaching assistants model specific ways in which they wanted the learner to eat. Again, this strategy was used for all children; however, teachers reported that this was often focused on older children in the study from around 7–9 years of age who were able to appropriately model feeding behaviours. For instance, the teachers described how they would sit down next to the learners and model appropriate mealtime behaviours, such as sitting on a chair, using a placemat and utensils to eat, as well as swallowing their food and not spitting it out. This process was described by Teacher 4:

‘So if you put food in front of him, he’ll just take it and throw it at you. I had to teach him not to do that and show him how to sit.’ (Teacher 4, age 31, English)

Teacher 3 also shared how she modelled specific eating techniques and encouraged the children to slow down during mealtime:

‘He didn’t eat neatly, so if he had a hotdog, he’ll try to push that whole hotdog into his face … urgh, into his mouth. So I had to teach show him how to eat slower.’ (Teacher 3, age 45, Sotho)

Teachers targeted specific mealtime behaviours that they felt important such as ‘eating neatly’, as reported by the teacher earlier. However, teachers also tried to be cognisant of the child’s different backgrounds when modelling behaviour as teachers were aware that children might have different mealtime rules, routines and rituals at home and with their families. Teachers handled this differently, with some trying to incorporate the child’s customs into mealtimes, while others did not. The prominent mealtime custom teachers spoke about was whether or not to let the children eat with their hands. Teacher 2 explained how she let a child eat with his hands, knowing this was his mode of eating at home:

‘It’s a challenge even still today but I’m taking it step by step and one by one because with our kids it’s not one fits all. I would let him use his hands because it’s something he does at home and it’s allowed and so it’s okay.’ (Teacher 2, age 27, Sotho)

However, teachers had different approaches to the ways in which they incorporated the child’s eating practices in the home and school environment, and what they were and were not willing to accept in the classroom:

‘If it is in the child’s culture to eat with their hands, I do try respect it but also try and teach them manners … that there is a little bit of a balancing to find … but we do try and respect cultures in general but the aim is certainly to let them eat. So I teach the children to eat with a spoon because … So I hold his hands and take the food from the plate and then to put in his mouth … so I first train him how to hold a spoon and it’s been difficult even until today. Others used to eat with spoons just this child only.’ (Teacher 1, age 31, isiZulu)

In the given excerpt, the teacher explains how she is aware that the child may eat with their hands at home, but she still feels that she would like all the children in her class to eat with spoons. The teacher then reports how she spends time modelling and ‘training’ the child to eat with a spoon to establish her own mealtime rules and rituals in the classroom. This is an important consideration for feeding in the classroom and the influence of the teacher on the ways in which feeding behaviour is being modelled.

Teachers utilising this strategy mentioned that this strategy was time-consuming and often required more time compared with when the child could eat independently, ‘and the time … it takes 15 min to 20 min if they feed themselves but 30 minutes when you are feeding them’ (Teacher 1, age 31, isiZulu). However, teachers stated that they were willing to put in this additional time as it would ensure the child is able to eat and fit in with the rest of the class regarding specific mealtime rituals and behaviours.

### Positive reinforcement

Five teachers in this study spoke of using positive reinforcement to enhance eating. Positive reinforcement was provided in different ways, such as through encouragement and verbal praise, as explained by Teacher 1, ‘And also lots and lots of prompting and encouraging and praises, hey well done … would you like to try again?’ Teacher 2 detailed a similar experience motivating a child to eat by using words of encouragement, ‘I encourage the child like “Hey try one more bite” and when they do, this is an achievement so you encourage that’. One teacher reported that when she changed her language to the child’s home language, this positively impacted the child’s acceptance and response to new foods (‘wenze kahle’ [*well done*]) (Teacher 8, age 34, isiZulu).

These verbal reinforcements were used to increase the acceptance of familiar foods the child was currently eating and in the introduction of new and unfamiliar foods. However, teachers reported that when using positive reinforcement to try new and unfamiliar foods, this strategy was less successful. Teachers reported that they struggled to encourage children in the class to eat unfamiliar foods. New and unfamiliar foods were often provided by the parents in the lunchbox when they themselves were trying new foods at home or if the child has been put on a new diet (such as gluten-free). This was described by Teacher 6:

‘And then if you see him wanting to put it in the bin, I would try and say, “hey, don’t do that, let’s give this a try.” He would actually start crying and screaming because he wants to throw it away now. So now if a child is taken off, if they are on maybe its gluten-free because of the weight, so they put the child on the diet but the child do not eat certain foods anymore so now they see his friend eating it or us as teachers are eating it then they get meltdowns or they don’t feel happy about it. They always like start grabbing things and not responding when you try and get them to eat their food.’ (Teacher 6, age 45, Afrikaans)

Teachers reported that positive reinforcement as a strategy did not always work and that it was often learner-dependent. Positive reinforcement was used to target food-related and behavioural problems in order to increase acceptance of more portion sizes, introduce new textures or encourage appropriate mealtime behaviour. In this study, positive reinforcement was used to target behaviours such as children refusing their food, throwing it or spitting it out:

‘[*I*]f they don’t want the food, they’ll spit it out or they’ll throw you with the food or throw it on the floor so that they don’t need to eat it.’ (Teacher 5, age 25, English)

In order to combat the child’s behaviour and food refusal, teachers would offer an object, preferred food item or activity that is of high value to the learner to encourage reoccurrence of a behaviour. This particular strategy was related to those teachers who were trained on applied behaviour analysis (ABA), and teachers using this method expressed confidence in using this strategy and one that they found to be highly successful, ‘ABA strategy where you kind of reward them with food. You reward them with a tiny piece of sweet or chip or something and it works’ (Teacher 4, age 31, English). Another teacher gave an example of how this strategy was used by allowing the child to play on the iPad:

‘So … I am ABA trained … so the ABA method of positive reinforcement … so small bite at a time … one reinforcement …one or two bites … reinforcement … that kind of thing is a strategy that I’ve been using … since my training which works quite well. Ya, so that positive … uhm having something that is of high value, not just a small value. It has to be of high value for the reinforcement to actually encourage them to do that. If the parents do want to introduce new textures … that is always the best method that I’ve used actually to help with that.’ (Teacher 5, age 25, English)

### Offering choices and alternatives

A novel theme that emerged in this study was spoken by two teachers, regarding offering choices and food alternatives as a strategy. One teacher described how she would place the lunchbox in front of the child and allow them to choose what they would like to eat, regardless of whether it may have been appropriate for that meal or not:

‘So he’ll decide what to eat. It doesn’t matter if he decide noodles for breakfast or Weetbix for lunch but as long as he eats but it’s not a problem, so long as the child has something to eat. So what we do is we put the lunchbox and we tell them, okay let’s see what you want to eat, you choose what you want to eat.’ (Teacher 3, age 45, Sotho)

The teacher went on to explain that she found this strategy to be very helpful as it reduced the child’s stress around mealtimes and eating and was also time-efficient, ‘no one is fighting, no one is angry and sometimes it’s because a simple thing that can take five minutes’. The teacher also expressed the value of this strategy to facilitate language and communication for those children who were non-verbal or who had limited expressive language. Offering the children choices functioned as an avenue to facilitate their expressive language abilities and communicative intent.

Another teacher spoke about offering food alternatives to ensure that the child would eat, even using her own money to purchase an alternative food option. Teacher 6 described how she had a child in her class who did not want to eat the food in his lunchbox and had a ‘meltdown’ and then refused to eat. The teacher was worried the child would not eat anything and ordered a familiar food item (pizza) from a particular restaurant that she knew the child enjoyed and would eat:

‘I had to take money out of my pocket for pizza because the learner wanted pizza. So … because the learner so clever, she only eats Debonairs [*pizza outlet*]. Because Debonairs is quite expensive. This is when I had to bell [*call*] Debonairs and to do a delivery … buy those small ones of R25.00 fresh from Debonairs.’ (Teacher 6, age 45, Afrikaans)

## Discussion

This qualitative study is one of the first to explore teachers’ implementation of management strategies when feeding children with autism in a South African classroom. Although the sample size was small, data saturation was achieved by focusing interview questions on a specific phenomenon. The study revealed that feeding and mealtimes presented challenges for teachers, irrespective of their years of teaching experience. These findings are consistent with studies examining the experiences of caregivers or mothers of children with autism, who also reported difficulties during mealtimes (Adams, Verachia & Coutts 2020; Adams et al. 2021; Ausderau & Juarez 2013; Gobrial 2018; Rogers, Magill-Evans & Rempel 2012). In previous studies on caregivers, they reported that mealtimes were difficult and often negatively impacted family mealtimes. This study provides some insights into the teachers’ experiences, some of which were similar across teachers and some unique. The study highlighted the different ways in which teachers tried to support each child’s feeding needs and the different strategies that were employed for different children. Unfortunately, as there is not a specific intervention that teachers are able to employ, many relied on trying to understand the child and their own knowledge and experience of working with children with autism and associated feeding difficulties.

Teachers in this study demonstrated varying levels of experience and training in working with children with autism. While some had received additional training on autism, many lacked guidance on supporting feeding in children with autism, and none had access to feeding specialists or SLTs at their schools. Nevertheless, all teachers stressed the significance of understanding each child in their class and their unique needs. This practice aligns with the common approach among teachers to familiarise themselves with the individual requirements of their students (Benson, Karlof & Siperstein 2008; Edwards & Mutton 2007; Sumbane et al. 2023; Syriopoulou-Delli, Cassimos & Polychronopoulou 2016). Observing and interacting with the children proved essential, particularly for those requiring additional support and accommodations in the classroom. Hence, providing these teachers with additional training becomes essential to better support the children and ensure improved feeding outcomes by implementing appropriate strategies and accommodations in the classroom.

When targeting feeding, teachers did not rely on one approach but used a combination to address either food-related behaviours or behavioural problems, with some of the strategies overlapping and addressing mealtime behaviours. Strategies used by the teachers were similar to those reported by caregivers (Adams et al. 2020; Ausderau et al. 2019; Farag et al. 2022). However, how teachers implemented the different strategies were slightly different as teachers were required to focus on more than one child during mealtimes. Furthermore, many of the strategies used were child-specific, dispelling the idea of a one-size-fits-all approach and reinforcing the importance of understanding each child’s individual needs (Bellomo et al. 2020). This technique required an investment of time on the part of the teacher to confirm what did or did not work for each child while incorporating aspects that they felt were appropriate for the child, such as changing their language of instruction. An interesting finding was that many teachers did not employ peer-to-peer techniques, which have shown success in previous studies (Padmanabhan & Shroff 2020) and could potentially aid in managing the time-consuming nature of the feeding strategies used. Teachers may be unaware of this or that there may not be appropriate behavioural models in the classroom as many children with autism will also have associated behavioural difficulties. Further exploration of peer-to-peer techniques may be warranted to improve feeding outcomes for children with autism.

Teachers found bolus modification to be the common and most successful strategy employed. This aligns with previous research suggesting bolus modification as an effective way to manage fussy eating in children with autism (Curtin et al. 2015). Another less common but effective strategy reported by teachers was offering choices, which not only improved the child’s autonomy and food acceptance but also facilitated language and communication, especially for children with limited expressive language skills. Further investigation into the success of offering choices in enhancing expressive language abilities and food acceptance in children with autism is warranted, as children were often only provided with a choice for one meal. Moreover, this approach may require parents to provide different food options for each meal, which could have financial implications that need careful consideration within the South African context.

Teachers are often faced with a dilemma when dealing with children who refuse their food. They must decide whether to allow the child to eat nothing or offer an alternative, even if it means providing unhealthy food options, just to ensure that the child consumes something. Many teachers prioritise ensuring that the children eat something, leading them to offer unhealthy alternatives inadvertently reinforcing fussy eating behaviours and creating negative associations with food (Kutbi 2020). Over time, this may result in a lasting aversion to certain foods and an increased risk of developing unhealthy eating habits. Therefore, it is crucial to distinguish between providing different food options that are healthier versus unhealthier, such as fast food. In the current study, it became apparent that some teachers might not fully grasp the potential impact of offering unhealthier or more preferred food choices. One teacher’s primary concern was ensuring the child would eat, to the extent of using her own money to purchase the food. However, it is essential to consider food choices and the financial implications of fast food options in South Africa. Previous research has highlighted the increased costs of fast foods for parents of children with autism, who provide such options just to ensure their child eats (Adams et al. 2020).

Teachers should be made aware of the different ways in which food options and alternatives can be provided in order to foster a more positive relationship with food, while still allowing for autonomy in the feeding process (Johnson et al. 2019; Ledford et al. 2018). By doing so, a healthier approach to mealtime can be cultivated, reducing the risk of reinforcing unhealthy eating behaviours in children with autism. Furthermore, teachers themselves should be knowledgeable about evidence-based practices in managing feeding difficulties for children with autism. While two teachers in the study were trained in ABA and employed ABA methods to manage feeding, research has indicated that ABA may result in physical and psychological trauma for children with autism and promote dependency (Sandoval-Norton, Shkedy & Shkedy 2019, 2021). Unfortunately, majority of the available treatment and interventions are not consistently aligned with evidence-based practices, and they are often not suitable for the South African context. Most of this information originates from high-income countries like the United States and the United Kingdom, which may not fully cater to the specific needs and circumstances of South Africa (Abubakar et al. 2016; Ruparelia et al. 2016; Soto et al. 2015). Therefore, it is essential to examine available treatment options and assess their feasibility and appropriacy in the South African and African context to ensure the development and utilisation of appropriate measures.

Mealtimes are deeply embedded in both culture and context. This study illustrated how teachers’ own beliefs and values were incorporated into classroom mealtime rituals, even if they were different from the children’s mealtime routines and rituals at home. Prior research has highlighted the importance of eating together as a symbol of unity and collective identity (Fiese 2021; Ochs & Shohet 2006; Tuomainen 2014). The study revealed that teachers felt it was important for their mealtime beliefs and values to be integrated into the classroom, such as ‘training’ the child to eat with utensils, despite this not being enforced at home. While accommodating every child’s mealtime routine and ritual may not always be feasible, especially in classrooms with diverse backgrounds, such as in South Africa, teachers should strive to be flexible and sensitive to each child’s culture, beliefs and values within the classroom.

## Conclusion

As autism prevalence rates increase, and more teachers can expect learners with autism in their classroom, it is important that we understand the ways in which feeding can be managed in the classroom. There were some limitations in the study. While the small sample size was imperative for capturing the experiences of teachers, this study was an exploration, and further research on this area is required. While the focus of this study was on the teacher report, in analysing the results, which offered valuable insights, the inclusion of parents, therapists and other school personnel would enhance the insights gained and help to broaden the scope of the findings. Future studies may want to provide a more comprehensive and holistic representation of teachers from other countries, backgrounds and contexts. The inclusion of observational data may be of great importance to better understand the teacher’s experiences and see what is happening in the classroom setting. Further research is necessary to develop evidence-based strategies that can effectively enhance the feeding management of children with autism in the classroom. These strategies can then be used to formulate specific guidelines and policies catering to the unique feeding needs of these children.

This study makes a valuable contribution to the global research and literature on teachers feeding children with autism in a South African classroom. It aims to expand the existing body of literature on children with autism and their associated feeding difficulties, focusing on diverse contexts, including low- and middle-income countries (LMICs) and Africa. The implications of this study underscore the importance of providing training for teachers working in special needs schools and the development of treatment methods that are culturally appropriate and responsive to the specific context. The findings emphasise the need for collaboration between teachers, caregivers and other healthcare professionals when working with school-aged children, especially those with disabilities. It highlights the significance of considering cultural and contextual variations in the school setting, while managing feeding difficulties. By addressing these aspects, the study contributes to improving the support and care provided to children with autism and feeding challenges in the educational environment.

## Footnotes

1 Samp is an African food made of dried corn kernels that have been pounded and chopped until broken.

## Appendix 1

### Interview schedule

1. Please tell me more about mealtimes and feeding in the classroom.
2. Can you explain how a typical feeding session goes between you and the child with autism?
3. What types of feeding difficulties have you experienced when feeding children with autism?
4. Did anyone prepare you in any way for the feeding difficulties that children with autism may present with? That is, did the parents provide you with any information regarding the child’s feeding?
5. Are there any strategies you are using to try and manage such feeding difficulties, and to what extent are they successful?
